# Oral pyridoxine can substitute for intravenous pyridoxine in managing patients with severe poisoning with isoniazid and rifampicin fixed dose combination tablets: a case report

**DOI:** 10.1186/s13104-017-2678-6

**Published:** 2017-08-08

**Authors:** M. D. S. A. Dilrukshi, C. A. P. Ratnayake, C. A. Gnanathasan

**Affiliations:** 1 University Medical Unit, National Hospital of Sri Lanka, Colombo 10, Sri Lanka; 2 Department of Clinical Medicine, University of Colombo, Colombo 10, Sri Lanka

**Keywords:** Isoniazid, Rifampicin, Poisoning, Antidote, Pyridoxine

## Abstract

**Background:**

Fixed drug combination of isoniazid and rifampicin is a rare cause of poisoning even in endemic countries for tuberculosis infection. Severe poisoning can cause severe morbidity and mortality if not treated promptly. Though intravenous pyridoxine is the preferred antidote for severe standard isoniazid poisoning it is not freely available even in best of care centers. We describe a case of severe poisoning with fixed drug combination of isoniazid and rifampicin successfully managed with oral pyridoxine at national hospital of Sri Lanka.

**Case presentation:**

A 22 year old, Sri Lankan female presented to a local hospital 1 h after self-ingestion of 28 tablets of fixed drug combination of isoniazid and rifampicin which contained 4.2 g of standard isoniazid and 7.2 g of rifampicin. One and half hours after ingestion she developed generalized tonic–clonic seizure with loss of consciousness. She was given intravenous diazepam 5 mg immediately and transferred to national hospital of Sri Lanka, for further care. Upon arrival to tertiary care hospital in 3.5 h of poisoning she had persistent vomiting, dizziness and headache. On examination, she was drowsy but arousable, orange–red discoloration of the body was noted even with the dark skin complexion. She also had orange–red colour urine and vomitus. Pulse rate was 104 beats/min, blood pressure 130/80 mmHg, respiratory rate was 20 breaths/min. The arterial blood gas analysis revealed compensated metabolic acidosis and mildly elevated lactic acid level. Considering the clinical presentation with neurological toxicity and the large amount of isoniazid dose ingested, crushed oral tablets of pyridoxine 4.2 g (equal to standard isoniazid dose ingested) administered immediately via a nasogastric tube since intravenous preparation was not available in the hospital. Simultaneously forced diuresis using intravenous 0.9% saline was commenced in order to enhance excretion of toxic metabolites via kidneys. She had no recurrence of seizures but had acute liver injury subsequently which gradually improved with supportive care. Her liver functions found to be completely normal 1 week after the discharge.

**Conclusions:**

Poisoning with fixed drug combination of isoniazid and rifampicin tablets is rare but can cause severe morbidity and mortality if not treated promptly. Oral pyridoxine can substitute for intravenous pyridoxine with almost similar efficacy at a low cost in managing patients with acute severe standard isoniazid poisoning in resource poor setting.

## Background

Isoniazid (standard INH) and rifampicin are first line anti-tuberculosis drugs. Rifampicin has bactericidal activity against *Mycobacterium tuberculosis* (TB) by inhibiting bacterial DNA-dependent RNA polymerase [1]. Isoniazid is a pro-drug activated by bacterial catalase–peroxidase and kills actively growing tubercle bacilli by inhibiting the biosynthesis of mycolic acids which are major components of cell wall of *M. tuberculosis* [1, 2].

Fixed drug combination of isoniazid and rifampicin (FDC-IR) used in all cases of tuberculosis as first line therapy. Despite the vast usage of FDC anti-TB therapy, poisoning with FDC-IR tablets is rarely reported in the literature which may be due to under reporting. However there are few reported cases on ingestion of isoniazid alone or in combination with rifampicin leading to severe morbidity and mortality from India but no cases reported from Sri Lanka [3]. Although rare, the outcome is considered to be extremely poor when the poisoning is severe and specific antidote is not readily available [3].

Pyridoxine is the antidote for severe isoniazid poisoning and there is no specific antidote identified for rifampicin toxicity. Intravenous pyridoxine is recommended in large doses when symptoms of acute neurotoxicity develop with Isoniazid poisoning [3–7]. Intravenous (IV) pyridoxine is not widely available even in best of care centers and there are few reported cases of isoniazid poisoning successfully managed with oral pyridoxine in the acute setting when IV preparation is not available [4, 8].

Severe FDC-IR poisoning immediately manifest as generalized tonic–clonic seizures, severe metabolic acidosis and subsequently lead to coma if not promptly treated [3]. We present a case of severe FDC-IR poisoning presented with generalized tonic–clonic seizures successfully treated with oral pyridoxine therapy in a tertiary care hospital of Sri Lanka.

## Case presentation

A 22 year old, previously healthy Sri Lankan female presented to the government hospital of Udupila, Sri Lanka, in September 2016, around 10.00 a.m., 1 h after self-ingestion of 28 tablets of FDC-IR which contained 4.2 g of standard INH and 7.2 g of rifampicin which was prescribed for her uncle, in an attempt of deliberate self-harm after a domestic dispute. The family had attempted induced emesis with water and coconut milk at home unsuccessfully.

On admission the patient had dizziness, nausea and vomiting. While being seeing in the medical ward at 10.30 a.m., she developed generalized tonic–clonic seizure which lasted about 3 min and associated with loss of consciousness. She was given IV diazepam 5 mg immediately. However gastric lavage and activated charcoal were not given due to reduced level of consciousness which was attributed to post ictal drowsiness. She was transferred to the national hospital of Sri Lanka immediately for further care.

**Fig. 1:**
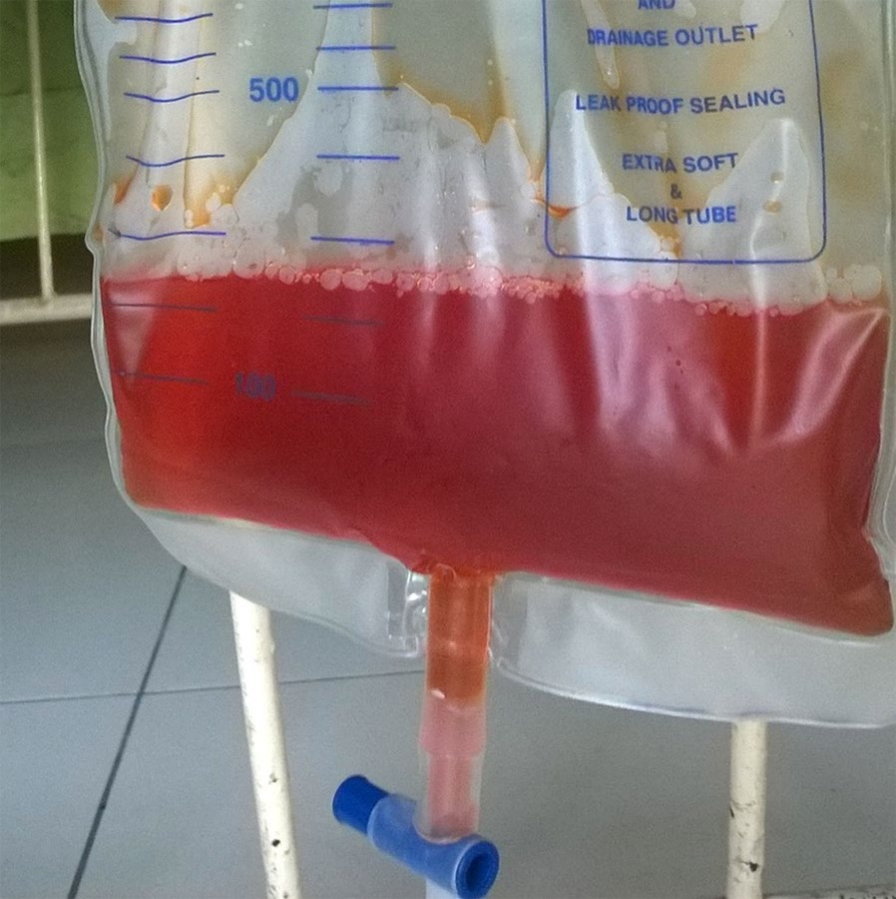
*Orange*–*red* colour urine in the urinary bag

She arrived to the national hospital around 12.30 p.m. (in 3.5 h of poisoning), with persistent vomiting, dizziness and headache. On examination, she was drowsy, but oriented and rational. No evidence of asterixis or coagulopathy. Orange–red discoloration of the face, hands and feet was noted even with the dark skin complexion. She also had orange–red colour urine (Fig. 1) and vomitus. Her pulse rate was 104 beats/min, regular and blood pressure 130/80 mmHg. The respiratory rate was 20 breaths/min, pulse oximetry of 98% on room air and auscultation of lungs normal. Abdomen and nervous system examination was unremarkable.

The arterial blood gas analysis was as follows; pH 7.36, HCO_3_-20 mmol/L, PCO_2_ 24 mmHg, pO_2_ 135 mmHg, Base excess-5, lactate level 2.3 and O_2_ saturation 100%. Considering the clinical presentation with neurological toxicity and the large amount of Isoniazid dose ingested, administration of antidote was decided. Since IV pyridoxine was not available at the hospital, crushed oral tablets of pyridoxine 4.2 g (equal to INAH dose ingested) administered immediately via a nasogastric tube and there were no recurrent seizures subsequently requiring repeated administration of pyridoxine. Simultaneously forced diuresis was commenced using IV 0.9% saline in order to enhance excretion of toxic metabolites via kidneys.

Her initial lab investigations revealed, white blood cell count of 14,870 (neutrophil 12,870); haemoglobin 12.3 g/dL; platelets 241,000; serum creatinine 58 µmol/L (normal 60–120 µmol/L); serum sodium 138 mmol/L (normal 135–148 mmol/L); potassium 3.9 mmol/L (3.5–5.1 mmol/L); aspartate transaminase (AST) 39 U/L (normal <5 U/L); alanine transaminase (ALT) 21 U/L (normal <35 U/L), total bilirubin 63 µmol/L (normal 5–21 µmol/L); prothrombin time (PT) 13.1 s; international normalizing ratio (INR) 1.03 and electrocardiogram was normal. Her liver functions started to deteriorate from Day 2 with persistent anorexia and vomiting and gradually started to improve by Day 7 of admission. Highest values of liver functions reported were, AST 161 U/L; ALT 215 U/L; PT 18.5 s; INR 1.42. Mild metabolic acidosis present on admission was completely corrected by Day 2. She was provided with supportive care for acute liver injury and she made complete recovery upon discharge on Day 10 of the hospital admission. Her liver functions found to be normal 1 week after the discharge.

## Discussion

Tuberculosis (TB) is a global health challenge, with 10.4 million new cases and accounting for 1.4 million deaths in 2015. More than 95% of TB deaths occur in low- and middle-income countries like Sri Lanka [9]. In 1994, the World Health Organization (WHO) recommended the use of fixed dose combination (FDC) tablets, each combining two or more anti-TB drugs in order to minimize development of resistant strains of mycobacteria as an important step of combating this global health issue [10].

Fixed drug combination of isoniazid and rifampicin (FDC-IR) is widely used as first line therapy for TB infection. Our patient had ingested 28 tablets of FDC-IR which contained isoniazid 150 mg and rifampicin 300 mg per each, equivalent to 60 mg/kg body weight of isoniazid and 120 mg/kg body weight of rifampicin.

Isoniazid is a hydrazide derivative of isonicotinic acid which is absorbed rapidly from gastrointestinal tract reaching peak levels within 1–2 h. The distribution volume is 0.6–0.7 L/kg. It is excreted rapidly within 24 h in subjects with a normal renal function. Metabolism takes place by enzymatic acetylation and hydrolysis is in liver. The plasma half-life is 0.5–1.6 h by fast acetylation and 2.5 h by slow acetylation [11]. Isoniazid is normally dosed at 5 mg/kg/day up to a maximum of 300 mg/day. Toxicity occurs with doses as low as 10–30 mg/kg with manifestations being nausea, vomiting, blurred vision, and slurred speech. A dose over 20 mg/kg may be associated with hallucination, recurrent seizures, metabolic acidosis, hypotension and coma. Death may occur at doses of over 50 mg/kg [11].

The metabolite of isoniazid, isoniazid hydrazone inhibits formation of pyridoxal-5 phosphate from pyridoxine by inhibiting pyridoxine phosphokinase enzyme competitively. The other metabolite of isoniazid such as hydrazine and hydrazide also inhibits pyridoxal-5 phosphate. Pyridoxal-5 phosphate is an essential cofactor in gamma amino butyric acid (GABA) synthesis which is an important inhibitory neurotransmitter in the central nervous system. Thus, the metabolite of isoniazid inhibits formation of pyridoxal-5 phosphate and ultimately decreases GABA production. The decrease in GABA is associated with seizures and other central nervous system manifestations [11–13]. Isoniazid inhibits conversion of lactate to pyruvate which in turn results in lactic acidosis. The seizures activity further aggravates lactate accumulation and increase lactic acidosis. Acetyl hydrazine metabolite of isoniazid is hepatotoxic. Sometimes, standard INH also causes hyperglycemia by blocking specific steps in Krebs cycle that requires nicotinamide adenine dinucleotide (NAD) and also from stimulating glucagon secretion. Thus, isoniazid toxicity manifests with central nervous dysfunction and hepatic dysfunction with metabolic abnormalities such as lactic acidosis, hyperglycemia, and hyperkalemia [11–13].

Rifampicin in toxic doses is known to produce gastrointestinal, hepatic, renal dysfunction, hematological, and central nervous system manifestations. It often presents with metabolic acidosis, convulsions, thrombocytopenia, cholestatic jaundice, oliguric renal failure, and red-man syndrome. The typical features of red man syndrome are glowing red discoloration of skin and periorbital odema. The toxicology findings are attributed to high concentration of rifampicin and two major metabolites, 25-desacetyl rifampicin and 3-formylrifamycin. The toxic effects have been described with ingestion of 9–12 and 14–15 g of rifampicin in various situation. About 70–80% of rifampicin is bound to protein and is distributed throughout body. The diacetyl metabolite is less toxic and its aqueous solubility results in better elimination in bile. In general, rifampicin is well tolerated by man even in very high doses and intoxication, with fatal outcomes being exceptional [14, 15].

Toxic over-dosage of Isoniazid and rifampicin in combination cause variety of manifestations of each drug toxicity such as convulsions, confusion, coma, metabolic acidosis, rhabdomyolysis, renal failure, and variable hepatotoxicity [16].

Our patient had ingested a large standard INH dose which has led to rapid development of CNS toxicity within 2 h of ingestion. Furthermore she has orange–red discoloration of the body, tears and urine indicating rifampicin overdose. She had compensated metabolic acidosis and marginally elevated lactic acid level.

Pyridoxine is considered to be the specific antidote in acute isoniazid poisoning which correct the GABA deficiency. The sooner pyridoxine is given the fewer the complications. For poisonings in which the amount of isoniazid ingested is known, pyridoxine is dosed on a milligram-for-milligram basis. It should be infused slowly by intravenous push at a rate of approximately 1 g/min [3–7]. However access for intravenous preparations of pyridoxine was not readily available during acute management according to the most reported cases [4]. Thus use of oral pyridoxine tablets as an alternative was encouraged in several case reports with promising results. Oral pyridoxine is absorbed within 20 min of administration, and peak plasma concentrations are rapidly achieved. Oral tablets are inexpensive and ubiquitously available [8].

Our patient was given oral pyridoxine immediately after admission to the National hospital and she didn’t develop further seizures. She was commenced on forced diuresis with 0.9% saline in order to augment excretion of the toxic metabolites via the kidneys and clearance of the acidosis. Though she developed biochemical evidence of acute hepatotoxicity she did not develop overt liver failure.

## Conclusions

Fixed drug combination of isoniazid and rifampicin tablet poisoning is a rarely reported cause of poisoning even in TB endemic countries which has severe morbidity and mortality if not treated promptly. Oral pyridoxine can substitute for IV pyridoxine with almost similar efficacy at a low cost in managing patients with acute severe standard INH poisoning in resource poor setting.

## Abbreviations

ALT: alanine aminotransferase
AST: aspartate aminotransferase
CNS: central nervous system
DNA: deoxyribonucleic acid
FDC-IR: fixed drug combination of isoniazid and rifampicin
GABA: gamma-aminobutyric acid
INAH: isoniazid
INR: international normalization ratio
IV: intravenous
NAD: nicotinamide adenine dinucleotide
PT: prothrombin time
RNA: ribonucleic acid
TB: tuberculosis
WHO: World Health Organization
